# Design, synthesis, and biological characterization of a potent STAT3 degrader for the treatment of gastric cancer

**DOI:** 10.3389/fphar.2022.944455

**Published:** 2022-08-12

**Authors:** Haobin Li, Lingling Wang, Fei Cao, Dehua Yu, Jing Yang, Xuefei Yu, Jinyun Dong, Jiang-Jiang Qin, Xiaoqing Guan

**Affiliations:** ^1^ The Cancer Hospital of the University of Chinese Academy of Sciences (Zhejiang Cancer Hospital), Institute of Basic Medicine and Cancer (IBMC), Chinese Academy of Sciences, Hangzhou, China; ^2^ School of Pharmaceutical Sciences, Zhejiang Chinese Medical University, Hangzhou, China; ^3^ School of Life Sciences, Tianjin University, Tianjin, China; ^4^ College of Pharmaceutical Science, Zhejiang University of Technology, Hangzhou, China; ^5^ Key Laboratory of Prevention, Diagnosis and Therapy of Upper Gastrointestinal Cancer of Zhejiang Province, Hangzhou, China

**Keywords:** stat3, gastric cancer, S3I-201, protac, degradation

## Abstract

Gastric cancer is a common malignant tumor that threatens human health, and its occurrence and development mechanism is a complex process involving multiple genes and multiple signals. Signal transducer and activator of transcription 3 (STAT3) has been elucidated as a promising target for developing anticancer drugs in gastric cancer. However, there is no FDA-approved STAT3 inhibitor yet. Herein, we report the design and synthesis of a class of STAT3 degraders based on proteolysis-targeting chimeras (PROTACs). We first synthesized an analog of the STAT3 inhibitor S3I-201 as a ligand, using the cereblon (CRBN)/cullin 4A E3 ligase ligand pomalidomide to synthesize a series of PROTACs. Among them, the SDL-1 achieves the degradation of STAT3 protein *in vitro*, and exhibits good anti-gastric cancer cell proliferation activity, inhibits invasion and metastasis of MKN1 cell, and induces MKN1 cell apoptosis and arrests cell cycle at the same time. Our study shows that SDL-1 is a potent STAT3 degrader and may serve as a potential anti-gastric cancer drug, providing ideas for further development of drugs for clinical use.

## 1 Introduction

Gastric cancer is one of the leading cause of cancer deaths all over the world (Brustugun, Møller, & Helland, 2014). The incidence of gastric cancer is reported to be highest in East Asia, followed by Central and Eastern Europe. In China, gastric cancer is one of the most common cancers causing over 374,000 deaths each year (Cao, Chen, Yu, Li, & Chen, 2021; Sung et al., 2021). Early gastric cancer can be treated with a variety of procedures, such as endoscopic resection and D1 or D2 lymphadenectomy (Li et al., 2019). However, patients with advanced gastric cancer often develop metastases, lose the opportunity for surgery, and switch to a drug-based approach. Due to the very limited selection of targeted drugs for gastric cancer, the development of targeted drugs for gastric cancer is imminent.

Signal transducer and activator of transcription 3 (STAT3), is an important oncogenic protein that promotes cancer cell proliferation and survival, as well as controls cell cycle progression and resists apoptosis (Wingelhofer et al., 2018). It is most frequently associated with poor prognosis in a variety of human cancers (Lai et al., 2015), including gastric cancer (Yu et al., 2022), melanoma (Swoboda et al., 2021) and breast cancer (Siersbæk et al., 2020), etc. Activation of STAT3 has been shown to be inversely correlated with patient prognosis (Rajendran et al., 2012; Liu et al., 2013). Therefore, STAT3 is regarded as an attractive target for gastric cancer therapy (Mali, 2015; Yu, Pardoll, & Jove, 2009). Over the past two decades, a large number of compounds have been identified that directly inhibit STAT3 activity through strategies such as virtual-based and high-throughput screening. However, no drugs have been approved to use in clinical practice (Jin et al., 2018).

Degrading STAT3 may represent another effective cancer treatment strategy (Hou et al., 2020; Dong, Cheng, Zhang, & Qin, 2021). Currently, a promising technology for inducing protein degradation is the proteolysis-targeting chimera (PROTAC) technology, which has become a hot research topic in the field of medicinal chemistry in recent years, providing a new therapeutic approach to address diseases driven by abnormal expression of disease-related proteins (Li et al., 2021; Neklesa, Winkler, & Crews, 2017; Yin et al., 2021). Traditional PROTACs are heterobifunctional molecules composed of three basic components: a ligand for binding the protein of interest (POI), a second ligand for binding and recruiting the E3 ligase degradation complex, and a linker that joins two ligands together. PROTACs bind to both POI and E3 ubiquitin ligase to form an E3-PROTAC-POI ternary complex that transfers ubiquitin to POI for polyubiquitination, leading to subsequent protein degradation via the 26S proteasome system (Li et al., 2021). Therefore, designing a PROTAC degrader of STAT3 as a new strategy to effectively target STAT3 may provide a feasible idea for the treatment of STAT3-related diseases.

Here, we described the synthesis and evaluation of a series of S3I-201-based PROTACs with cellular potency in gastric cancer cell lines. Our data showed that SDL-1 induced STAT3 protein degradation while inhibiting the growth activity of gastric cancer cells *in vitro*, suggesting that it could be used as a potential anti-gastric cancer drug and providing a new strategy for gastric cancer therapy.

## 2 Materials and methods

### 2.1 Data acquisition and analysis

Both the RNAi and the CRISPR dependency data for gastric cancer cell lines were downloaded from depmap portal (https://depmap.org/portal/) (Dempster et al., 2021). The TPM values for STAT3 RNA expression of TCGA and GTEx samples and survival data of TCGA STAD patients were downloaded from UCSC Xena (https://xena.ucsc.edu/) (Goldman et al., 2020). The STAT3 expression difference between tumor and normal/paratumor tissues was compared using the *t*-test. Then the patients were further separated by median of STAT3 expression levels. Survival analysis was done by Graphapd Prism and Logrank test was performed for the statistical analysis. *p* < 0.05 was considered as significant.

### 2.2 Chemistry

General information. All starting chemicals were purchased from commercial sources and all agents purchased were used without further purification. Anhydrous solvents were dried according to standard methods. Proton nuclear magnetic resonance (^1^H NMR) was recorded on a Bruker AVANCE NEO 400 spectrometer using deuterated chloroform (CDCl_3_) and deuterated methanol (CD_3_OD) as solvent. The residual solvent signal (CD_3_OD: ^1^H NMR: 3.31 ppm, CDCl_3_: ^1^H NMR: 7.26 ppm) was used for calibration referred to tetramethylsilane. Tetramethylsilane (TMS) was used as an internal standard and chemical shifts were expressed in δ units (ppm). The multiplicity of each signal is expressed as s (singlet), d (doublet), t (triplet), m (multiplet), brs (broad singlet). The progress of the reaction was monitored by spotting using thin layer chromatography (TLC, silica GF254) and observing with UV light (*λ* = 254 or 365 nm). Silica gel (200–300 mesh) was added to the column for separation and purification by column chromatography. High Resolution Mass Spectrometry (HRMS) was obtained on an Orbitrap Exploris 480.

Synthesis of Ethyl 2-(tosyloxy)acetate(3). Dissolve ethyl glycolate(1) and p-toluenesulfonyl chloride(2) in tetrahydrofuran (THF) in a 1:1 equivalent, add triethylamine dropwise at 0 °C. When TLC indicated the reaction was complete, add water to separate the phases, and use ethyl acetate extract 3 times. The organic layer solvents were combined, dried over anhydrous Na_2_SO_4_, filtered to obtain a filtrate, and the filtrate was concentrated under reduced pressure to obtain a crude product, which was subjected to flash column chromatography using n-hexane/ethyl acetate (3:1) as an eluent to obtain product 3 in 75% yield as colorless oil.

Synthesis of 2-(tosyloxy)acetic acid(4). The solution of compound 3 in ethanol was treated with 5% NaOH solution, the reaction system was stirred at room temperature for 3 h, the ethanol was removed under reduced pressure, and the pH was adjusted with 5% HCl until a large amount of white solid precipitated, filtered with suction, and dried to obtain the product 4 in 68% yield.

Synthesis of 2-chloro-2-oxoethyl 4-methylbenzenesulfonate(5). A mixture of compound 4 and thionyl chloride (SOCl_2_) was stirred and refluxed for 2 h at 80°C. Excess SOCl_2_ was removed under vacuum, and the product 5 could be used in the next reaction without further purification.

Synthesis of 4-(2-(tosyloxy)acetamido)benzoic acid(6). Compound 5 was dissolved in THF, added dropwise the solution of p-aminobenzoic acid (1 equiv) and triethylamine (3 equiv) in THF at 0°C, then returned to room temperature and continued to stir overnight. After the reaction was completed, it was concentrated under reduced pressure, washed with Na_2_CO_3_ solution, and adjust the pH with concentrated HCl until a large amount of white solid precipitated, filtered with suction, and dried to obtain the product 6 in 78% yield.

Synthesis of 2-(2,6-dioxopiperidin-3-yl)-4-fluoroisoindoline-1,3-dione(8). A mixture of 3-fluorophthalic anhydride (7), 3-aminopiperidine-2,6-dione hydrochloride and NaOAc (1:1:3) was dissolved in HOAc and the resulting mixture was stirred at 100°C for 16 h. The solution was concentrated under reduced pressure to obtain a residue after the reaction was completed, which was diluted with aqueous NaHCO_3_ and extracted three times with ethyl acetate. The combined organic phases were washed with aqueous NaHCO_3_ and brine, dried over anhydrous Na_2_SO_4_, filtered and concentrated to give the product 8 was an off-white solid and was used in the next step without further purification.

Synthesis of tert-butyl (2-((2-(2,6-dioxopiperidin-3-yl)-1,3-dioxoisoindolin-4-yl)amino)ethyl)carbamate(9a). Compound 8, tert-butyl (2-aminoethyl)carbamate and DIPEA (1:1:2) were stirred in DMF at 80 °C for 12 h. The mixture was diluted with water, extracted with ethyl acetate, washed with brine, dried over anhydrous Na_2_SO_4_, filtered, concentrated and purified to obtain 9a. A series of compounds 9a-9h were obtained in a yield of 60–80%.

Synthesis of 4-((2-aminoethyl)amino)-2-(2,6-dioxopiperidin-3-yl)isoindoline-1,3-dione(10a). Compound 9a was dissolved in DCM, trifluoroacetic acid (TFA) was added, and the reaction was carried out at room temperature for 2 h to obtain the deprotected compound 10a. The product 10a was used in the next step without further purification.

Synthesis of 2-((4-((2-((2-(2,6-dioxopiperidin-3-yl)-1,3-dioxoisoindolin-4-yl)amino)ethyl)carbamoyl)phenyl)amino)-2-oxoethyl 4-methylbenzenesulfonate(11a). S3I-201 analog 6 and condensing agent HATU were activated in DCM for 1 h, and then, DIPEA was added, the reaction was continued for 10 min. Finally, the solution of deprotection compound 10a in DCM was added. The reaction was carried out overnight at room temperature, extracted with water and DCM, the organic layer was dried over anhydrous Na_2_SO_4_, filtered and concentrated under reduced pressure to remove excess solvent. The obtained crude product was separated and purified by column chromatography with gradient elution to obtain the final product PROTAC 11a.

### 2.3 Molecular docking

The molecular docking studies was carried out by Autodock 4.2.6. The crystal structure of CRBN (PDB ID: 4CI3) was selected for the docking studies and retrieved from the RCSB Protein Data Bank (PDB, https://www.rcsb.org/). The 3D structure of pomalidomide was constructed by ChemDraw 20.0. 40 grid points, with 0.375 Å of spacing, were set in each dimension. The center of ligand binding region in the crystal structure was set as the center of grid box. The search grids corresponding to CRBN binding sites are identified as center_x, -6.944; center_y, 1.94 and center_z, -11.225; dimensions were: size_x, 40; size_y, 40 and size_z, 40. After the protein and compound were pretreated separately, the interaction on the receptor and compounds were studied and visualized by PyMOL 2.5.

### 2.4 Cell lines, and cell culture

Human gastric cancer cell line MKN1 was purchased from American Type Culture Collection (ATCC, Rockville, MD, United States). MKN1 cells were cultured in RPMI 1640 medium (Gibco/Life Technologies, Darmstadt, Germany) supplemented with 10% fetal bovine serum (FBS) at 37°C.

### 2.5 Cell viability assay

As described before (Hong et al., 2022), cells were seeded at 3,000 cells per well in 96-well plates overnight. Then the cells were exposed to indicated compounds at concentrations of 1, 10, 100 μΜ. Cell viability was measured after 72 h with addition of CCK8 reagent (Biosharp, Anhui, China). The IC_50_ value was calculated using non-linear regression analysis using Graphpad Prism.

### 2.6 Cell cycle assay

To determine cell cycle distribution, 5 × 10^5^ cells were plated in a 6 cm dish and treated with DMSO or indicated concentrations of SDL-1 for 24 h. Then cells were harvested and fixed with 95% ethanol at 4°C for more than 2 h. The fixed cells were incubated in PBS with PI (propidium iodide) and RNase (BD Pharmingen, United States). Cell cycle was determined by flow cytometry LSRFortessa (BD Bioscience, United States) and further analyzed by ModFit LT software (Verity Software House, Switzerland) as described previously (Yu et al., 2022).

### 2.7 Cell apoptosis assay

Referring to previously reported methods (Wang et al., 2020), 3 × 10^5^ cells were cultured in six-well plates and then treated with indicated concentrations of SDL-1 for 48 h. The treated cells were re-suspended with Annexin V-FITC and PI from FITC Annexin V Apoptosis Detection Kit I (BD Pharmingen, United States), and incubated at room temperature for 15 min. The early and late apoptosis rate of SDL-1 on cells were analyzed through the flow cytometer CytoFLEX LX (Beckman Coulter, United States).

### 2.8 Wound healing assay

Wound healing assay was performed as described previously (Qi et al., 2022). Cells grown in monolayers were gently scratched with a 10 μL pipette tip in the central region of cell growth, and then exposed to indicated concentrations of SDL-1 for indicated times. Images of cells were captured at indicated times after scratching to evaluate the effect of SDL-1 on cell migration.

### 2.9 Transwell assays

For the transwell invasion assay, cells (5 × 10^4^ cells/well) in serum-free medium were seeded into Boyden chamber (Corning, United States), and then treated with SDL-1 for 24 h. Cells that had penetrated through the pores were stained with Crystal Violet Stain solution 2.5% (Solarbio, China). Invaded cells were photographed by inverted microscope (Olympus CKX53).

### 2.10 Western blot

Western blot experiments were performed as previously reported (Xue et al., 2017; Qin et al., 2018). In brief, 3 × 10^5^ cells were cultured in 6-cm dishes treated with specific concentrations of SDL-1 (0, 10, 20, and 40 μM) for 24 h. And then all cells were lysed in RIPA lysis buffer (Absin Bioscience Inc., Shanghai, China) containing protease inhibitor mixture (PMSF). The cell lysates were quantified by the BCA Protein Assay Kit (Absin, Shanghai, China). Equal amounts of protein were resolved by SDS-PAGE and transferred onto nitrocellulose membranes (Bio-Rad, Hercules, CA, United States). Block the membranes in 5% skim milk at room temperature and incubated with primary antibody overnight at 4 °C followed by incubation with appropriate secondary antibody at room temperature. Antibodies used are as follows: STAT3 antibody (Cell Signaling Technology, #12640S), p-STAT3 antibody (Cell Signaling Technology, #49081S), and GADPH antibody (Proteintech, #60004-1-Ig). The protein bands were detected by ECL Chemiluminescent Substrate Reagent Kit (Biosharp, Hefei, China) and scanned using the ImageQuant 800 (Amersham, United Kingdom).

### 2.11 Statistical analysis

All data are presented as mean ± SEM for the three replicates and plotted using Graphpad Prism software (version 9.0). Differences between treatment and control groups were compared by *t*-test. *p* < 0.05 indicates statistical significance.

## 3 Results

### 3.1 STAT3 is overexpressed and associated with a poor prognosis in gastric cancer

**FIGURE 1:**
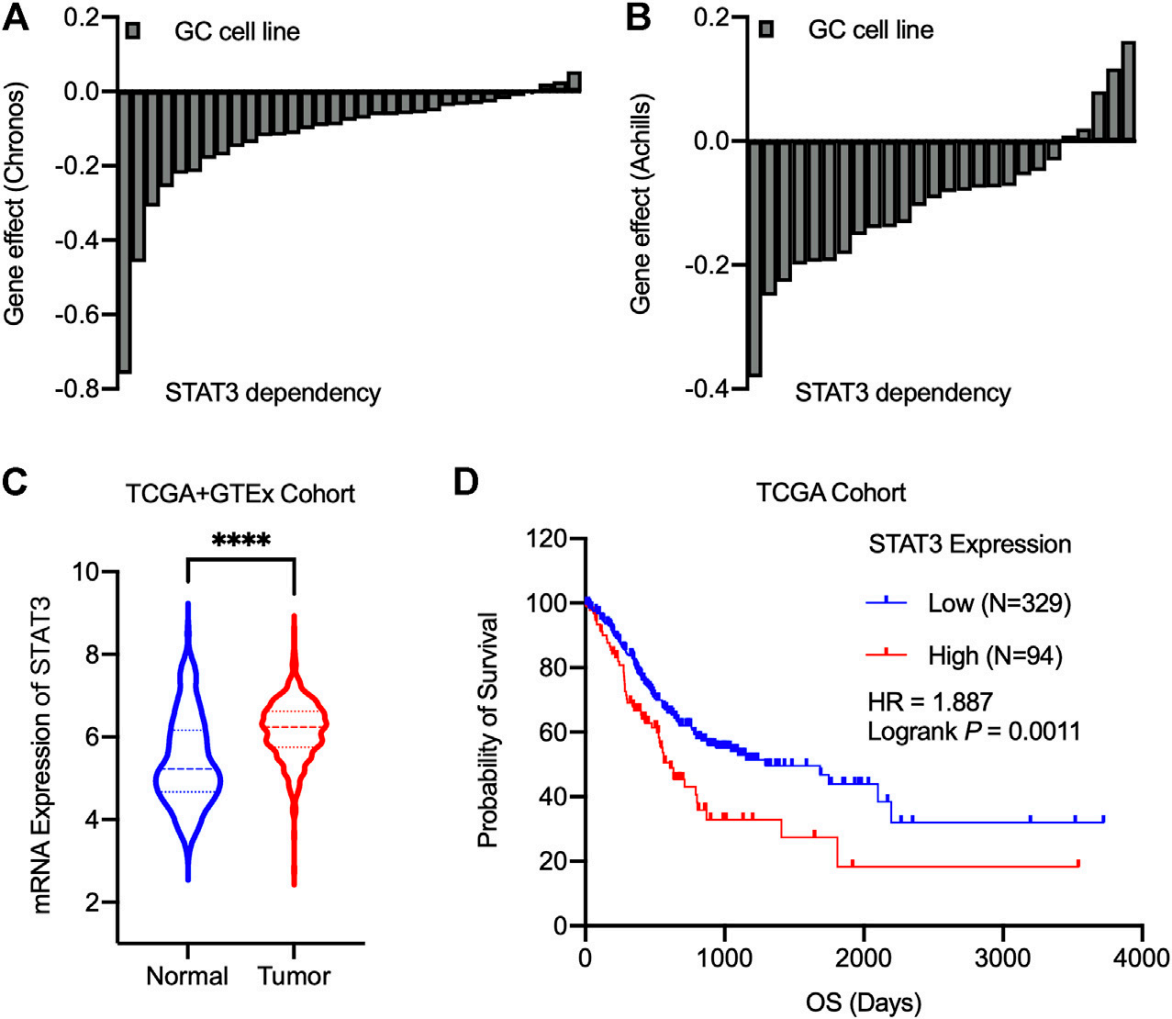
STAT3 serves as an essential gene for gastric cancer, and its overexpression correlates with poor prognosis. **(A,B)** The Chronos dependency score using CRISPR/Cas9 **(A)** and Achilles dependency score using RNAi **(B)** from cancer cell lines. A score of 0 indicates a gene is not essential, and a lower score indicates a higher likelihood that the gene is essential in a given cell line. **(C)** mRNA Expression levels of STAT3 for the normal and tumor samples. Statistics analysis was done using unpaired Student’s *t*-test. ****p < 0.0001. **(D)** TCGA survival analysis for gastric cancer patients based on STAT3 expression levels. Logrank test was performed for the statistical analysis.

We *in silico* analyzed the essentiality of STAT3 for the growth of gastric cancer cell lines identified by Chronos’ and Achilles’ projects (Figures 1A,B). Our results showed that almost gastric cancer cell lines exhibited growth modulation related to STAT3 expression. Further analysis found that at mRNA Level, STAT3 was highly expressed in malignant gastric cancer cells compared with normal and adjacent non-tumorous samples in TCGA and GTEx cohort (Figure 1C). Then TCGA gastric cancer survival data were divided into high expression and low expression groups based on the median value of STAT3 expression. Figure 1D revealed that higher RNA expression of STAT3 correlates with poor survival outcomes, suggesting that STAT3 may be a promising target for gastric cancer therapy.

### 3.2 Design of PROTAC STAT3 degraders

**FIGURE 2:**
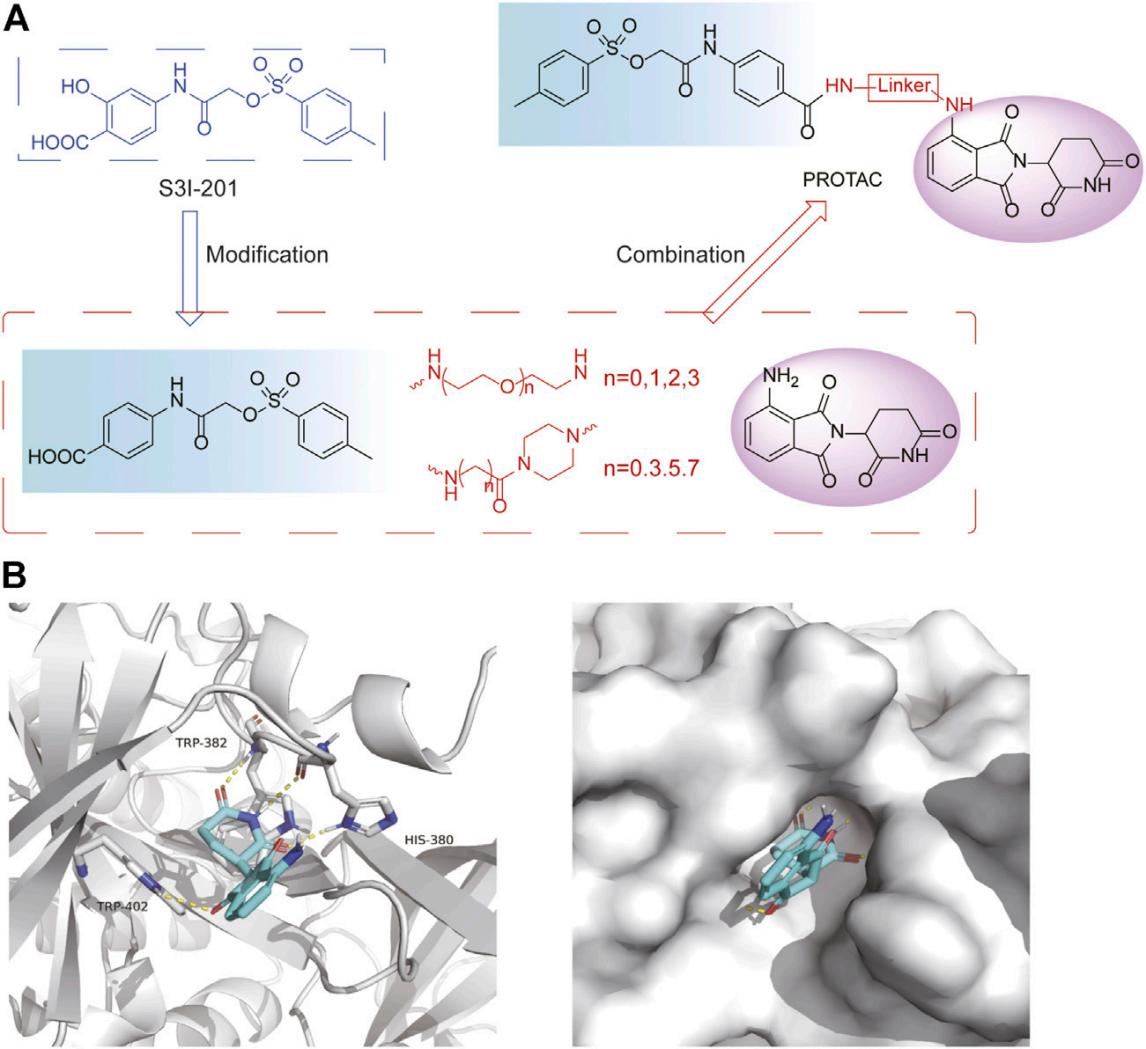
Design ideas a of PROTACs. **(A)** Design ideas of PROTACs. Using S3I-201 analog and pomalidomide as ligands, a series of STAT3 PROTACs were designed by changing Linker. **(B)** Docking model of pomalidomide binding to CRBN (PDB:4CI3). Pomalidomide is colored by atom type and CRBN is represented in cartoon form. Critical residues are shown as sticks. Hydrogen bonds are represented by yellow dashed lines.

PRTOAC molecules consist of target protein ligands, E3 ubiquitin ligase ligands and intermediate linkers. To design and synthesize PROTACs targeting STAT3, we made structural modifications based on the STAT3 inhibitor S3I-201, assuming that its carboxyl moiety might be the solvent-exposed region of linkers of different lengths (Figure 2A). Immunomodulatory drugs pomalidomide is a widely used cereblon (CRBN) ligand whose glutarimide moiety is considered as the major pharmacophore. According to the reported co-crystal structure (Fischer et al., 2014), the pomalidomide glutarimide carbonyl and the intermediate amide are hydrogen-bonded to the CRBN residues His 380 and Trp 382, respectively. The delocalized lone pair links the glutarimide nitrogen to the two glutarimide carbonyl groups and is coplanar with Trp 382. The opposing aliphatic face of the glutarimide ring is in close van der Waals contact with the hydrophobic pockets lined by Trp 382, Trp 388, and Trp 402 (Figure 2B). Besides, pomalidomide binds CRBN with high affinity (*K_d_* = 157 nM), superior to its analogs lenadomide (*K_d_* = 178 nM) and thalidomide (*K_d_* = 250 nM). Based on this, we selected pomalidomide as an E3 ubiquitin ligase ligand for PROTAC synthesis.

### 3.3 Synthesis of PROTAC STAT3 degraders

**FIGURE 3:**
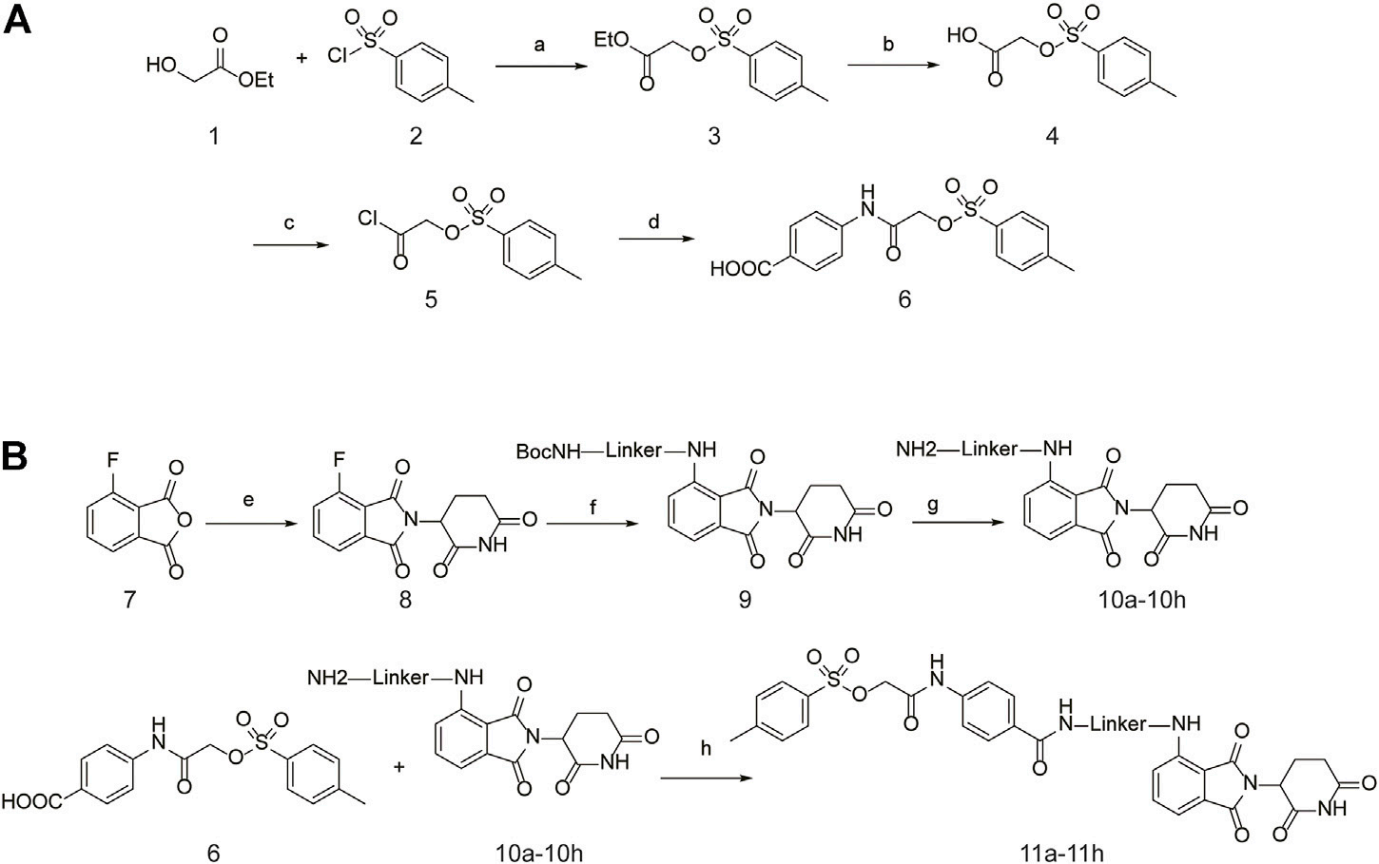
Synthetic route of PROTACs. **(A)** Synthesis of S3I-201 analogs. **(B)** Synthetic of PROTACs. Reagents and conditions: (a) THF, 0°C, 3 h; (b) 5% NaOH aq, EtOH, rt, 3 h; (c) SOCl_2_, 80°C, reflux, 2 h; (d) THF, Et_3_N, 0°C, overnight, rt; (e) 3-Aminopiperidine-2,6-dione hydrochloride, CH_3_COONa, CH_3_COOH, 100°C, 16 h; (f) BocNH-Linker-NH_2_, DIPEA, DMF, 80°C; (g) DCM, CF_3_COOH, rt, 2 h; (H) HATU, DIPEA, DCM, rt, 12 h.

The specific synthetic routes are as follows (Figure 3A): commercially available ethyl glycolate 1 and p-toluenesulfonyl chloride 2 are substituted and hydrolyzed to obtain 2-(tosyloxy)acetic acid (4). Intermediate 4 is subjected to acid chlorination and condensation to obtain S3I-201 analog 6. In Figure 3B, we described the linkage of the CRBN ligand pomalidomide to linker and the synthesis of PROTACs. Commercially available compounds 3-Fluorophthalic anhydride (7) and 3-Aminopiperidine-2,6-dione hydrochloride were subjected to a series of hydrolytic ring opening, nucleophilic substitution and deprotection to obtain compounds 10a–10 h. PROTAC compounds 11a-11 h were obtained by the condensation of compounds 6 and 10 by changing the length and chemical composition of the linker.

11a (SDL-1) 2-((4-((2-((2-(2,6-dioxopiperidin-3-yl)-1,3-dioxoisoindolin-4-yl)amino)ethyl)carbamoyl)phenyl)amino)-2-oxoethyl 4-methylbenzenesulfonate: yellow solid; ^1^H NMR (400 MHz, Chloroform-d) δ 8.05 (s, 1H), 8.00 (d, J = 8.6 Hz, 2H), 7.79 (dd, J = 8.4, 2.3 Hz, 3H), 7.57 (d, J = 8.7 Hz, 2H), 7.35 (d, J = 8.1 Hz, 4H), 6.89 (s, 1H), 6.41 (s, 1H), 4.85 (dd, J = 12.1, 5.4 Hz, 1H), 4.51 (s, 2H), 3.62 (s, 2H), 3.54 (d, J = 5.8 Hz, 2H), 2.88–2.64 (m, 4H), 2.40 (s, 3H), HRMS calcd for (M + H)+: 648.1764 found 648.1818.

11b 2-((4-((2-(2-((2-(2,6-dioxopiperidin-3-yl)-1,3-dioxoisoindolin-4-yl)amino)ethoxy)ethyl)carbamoyl)phenyl)amino)-2-oxoethyl 4-methylbenzenesulfonate: yellow solid; ^1^H NMR (400 MHz, Methanol-d4) δ 8.06–8.02 (m, 1H), 7.89 (s, 1H), 7.86 (s, 1H), 7.72 (d, J = 2.0 Hz, 1H), 7.71 (d, J = 2.2 Hz, 1H), 7.55 (s, 1H), 7.52 (d, J = 6.3 Hz, 1H), 7.44 (s, 1H), 7.42 (s, 1H), 7.07 (d, J = 8.5 Hz, 1H), 6.97 (d, J = 7.1 Hz, 1H), 4.97 (dd, J = 12.8, 5.5 Hz, 1H), 4.66 (s, 2H), 3.73 (t, J = 5.1 Hz, 2H), 3.69 (d, J = 5.5 Hz, 2H), 3.57 (t, J = 5.6 Hz, 2H), 3.50 (d, J = 3.1 Hz, 2H), 3.16–2.97 (m, 4H), 2.41 (s, 3H), HRMS calcd for (M + H)+: 692.2026 found 692.2005.

11c 2-((4-((2-(2-(2-((2-(2,6-dioxopiperidin-3-yl)-1,3-dioxoisoindolin-4-yl)amino)ethoxy)ethoxy)ethyl)carbamoyl)phenyl)amino)-2-oxoethyl 4-methylbenzenesulfonate; yellow solid. ^1^H NMR (400 MHz, Methanol-d4) δ 7.88 (d, J = 1.8 Hz, 1H), 7.86 (s, 1H), 7.72 (d, J = 2.1 Hz, 1H), 7.71 (d, J = 2.0 Hz, 1H), 7.49 (d, J = 1.6 Hz, 1H), 7.48–7.47 (m, 1H), 7.46 (d, J = 3.7 Hz, 1H), 7.44–7.43 (m, 1H), 7.42 (s, 1H), 6.97 (t, J = 7.5 Hz, 2H), 5.00 (dd, J = 12.7, 5.4 Hz, 1H), 4.63 (s, 2H), 3.72 (d, J = 5.3 Hz, 2H), 3.67 (d, J = 5.2 Hz, 2H), 3.56 (dd, J = 5.8, 4.0 Hz, 4H), 3.41 (t, J = 5.3 Hz, 2H), 2.85–2.59 (m, 4H), 2.41 (s, 3H), 2.09–2.03 (m, 2H), HRMS calcd for (M + H)+: 736.2289 found 736.2349.

11d 2-((4-((2-(2-(2-(2-((2-(2,6-dioxopiperidin-3-yl)-1,3-dioxoisoindolin-4-yl)amino)ethoxy)ethoxy)ethoxy)ethyl)carbamoyl)phenyl)amino)-2-oxoethyl 4-methylbenzenesulfonate: yellow solid. ^1^H NMR (400 MHz, Methanol-*d*_4_) δ 7.87 (d, *J* = 2.0 Hz, 1H), 7.85 (d, *J* = 2.2 Hz, 1H), 7.76 (d, *J* = 2.0 Hz, 1H), 7.74 (d, *J* = 2.1 Hz, 1H), 7.55 (d, *J* = 2.1 Hz, 1H), 7.53 (d, *J* = 2.2 Hz, 1H), 7.44 (s, 1H), 7.42 (s, 1H), 7.01 (d, *J* = 7.7 Hz, 3H), 5.05 (d, *J* = 5.7 Hz, 1H), 4.63 (s, 2H), 3.66–3.62 (m, 12H), 3.54 (d, *J* = 5.3 Hz, 2H), 3.42 (t, *J* = 5.2 Hz, 2H), 2.84–2.66 (m, 4H), 2.40 (s, 3H), HRMS calcd for (M + H)+: 780.2551 found 780.2520.

11e 2-((4-(4-(2-(2,6-dioxopiperidin-3-yl)-1,3-dioxoisoindolin-4-yl)piperazine-1-carbonyl)phenyl)amino)-2-oxoethyl 4-methylbenzenesulfonate: yellow solid. ^1^H NMR (400 MHz, Chloroform-d) δ 8.06 (s, 1H), 7.79 (dt, J = 8.6, 2.0 Hz, 2H), 7.61–7.50 (m, 3H), 7.37 (dt, J = 10.7, 6.2 Hz, 5H), 7.10 (d, J = 8.4 Hz, 1H), 5.23 (s, 1H), 4.89 (dd, J = 11.8, 5.3 Hz, 1H), 4.49 (s, 2H), 3.79 (d, J = 112.1 Hz, 4H), 3.28 (s, 4H), 2.93–2.55 (m, 4H), 2.41 (s, 3H), HRMS calcd for (M + H)+: 674.1921 found 674.1910.

11f 2-((4-((4-(4-(2-(2,6-dioxopiperidin-3-yl)-1,3-dioxoisoindolin-4-yl)piperazin-1-yl)-4-oxobutyl)carbamoyl)phenyl)amino)-2-oxoethyl 4-methylbenzenesulfonate: yellow solid. ^1^H NMR (400 MHz, Methanol-d4) δ 7.86 (d, J = 8.4 Hz, 2H), 7.78 (d, J = 8.8 Hz, 2H), 7.71–7.64 (m, 1H), 7.58 (d, J = 8.8 Hz, 2H), 7.46–7.38 (m, 3H), 7.29 (d, J = 8.3 Hz, 1H), 5.10 (dd, J = 12.5, 5.5 Hz, 1H), 4.66 (s, 2H), 3.80–3.74 (m, 4H), 3.71 (d, J = 6.6 Hz, 2H), 3.50–3.41 (m, 2H), 3.34 (t, J = 5.0 Hz, 2H), 3.28–3.25 (m, 2H), 2.77 (dd, J = 4.3, 2.5 Hz, 4H), 2.54 (t, J = 7.3 Hz, 2H), 2.40 (s, 3H), HRMS calcd for (M + H)+: 759.2448 found 759.2440.

11g 2-((4-((6-(4-(2-(2,6-dioxopiperidin-3-yl)-1,3-dioxoisoindolin-4-yl)piperazin-1-yl)-6-oxohexyl)carbamoyl)phenyl)amino)-2-oxoethyl 4-methylbenzenesulfonate: yellow solid. ^1^H NMR (400 MHz, Methanol-*d*_4_) δ 7.86 (d, *J* = 8.4 Hz, 2H), 7.75 (d, *J* = 8.7 Hz, 2H), 7.70–7.65 (m, 1H), 7.54 (d, *J* = 8.7 Hz, 2H), 7.41 (dd, *J* = 11.1, 7.6 Hz, 3H), 7.28 (d, *J* = 8.3 Hz, 1H), 5.11 (dd, *J* = 12.5, 5.5 Hz, 1H), 4.64 (s, 2H), 3.76 (q, *J* = 8.4, 6.2 Hz, 4H), 3.43–3.37 (m, 2H), 3.26 (t, *J* = 5.2 Hz, 2H), 2.93–2.80 (m, 2H), 2.78–2.69 (m, 2H), 2.47 (t, *J* = 7.4 Hz, 2H), 2.41 (s, 3H), 2.24–2.13 (m, 2H), 1.70 (d, *J* = 7.3 Hz, 2H), 1.65 (d, *J* = 7.2 Hz, 2H), 1.63–1.55 (m, 2H), HRMS calcd for (M + H)+: 787.2761 found 787.2728.

11h 2-((4-((8-(4-(2-(2,6-dioxopiperidin-3-yl)-1,3-dioxoisoindolin-4-yl)piperazin-1-yl)-8-oxooctyl)carbamoyl)phenyl)amino)-2-oxoethyl 4-methylbenzenesulfonate: yellow solid. ^1^H NMR (400 MHz, Methanol-*d*_4_) δ 7.88–7.84 (m, 2H), 7.77–7.73 (m, 2H), 7.68 (dd, *J* = 8.4, 7.2 Hz, 1H), 7.56 (dd, *J* = 8.8, 1.9 Hz, 2H), 7.41 (dd, *J* = 10.9, 7.6 Hz, 3H), 7.31 (d, *J* = 8.3 Hz, 1H), 5.11 (dd, *J* = 12.5, 5.5 Hz, 1H), 4.64 (s, 2H), 3.76 (d, *J* = 6.8 Hz, 4H), 3.39–3.33 (m, 4H), 2.81 (s, 2H), 2.78–2.70 (m, 2H), 2.44 (t, *J* = 7.6 Hz, 2H), 2.41 (s, 3H), 2.21–2.10 (m, 2H), 1.71–1.44 (m, 10H), HRMS calcd for (M + H)+: 815.3074 found 815.3140.

### 3.4 Anti-proliferation activity of compounds 11a–11 h against gastric cancer cells

In order to investigate the effects of eight compounds on the cell viability, four gastric cancer cell lines were treated with in increasing concentrations of compounds (1–100 μΜ). As shown in Table 1, we found that the all eight compounds had inhibitory effects on the viability of gastric cancer cells. Compared with positive control compound S3I-201, 11a (SDL-1) showed better anti-gastric cancer effects with the IC_50_ values of 31.52 , 26.49 , 11.78 , and 44.90 μΜ in HGC27, MGC803, AZ521, and MKN1 cells, respectively, consistent with the conclusion that in contrast to small molecule inhibitors (SMIs) that require occupation of the STAT3 binding site, PROTACs catalyze the degradation of multiple POI molecules through an event-driven pharmacological mechanism of action, exhibiting significantly lower concentrations than SMIs to trigger the required (Khan et al., 2020).

**TABLE 1:** Investigating the effect of linkers on the antiproliferative activity of STAT3 PROTACs towards four human gastric cell lines (HGC27, MGC803, AZ521, and MKN1).

Compd	Linker	IC_50_ (μM)			
		HGC27	MGC803	AZ521	MKN1
11a (SDL-1)	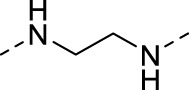	31.52	26.49	11.78	44.90
11b	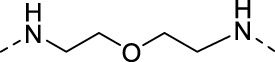	48.05	21.69	37.46	>100
11c	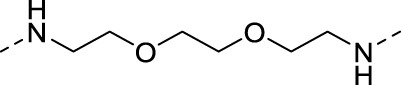	51.68	15.93	14.05	81.73
11d	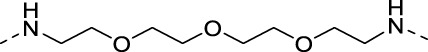	136.7	22.79	41.01	>100
11e	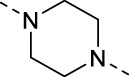	44.91	19.92	16.7	36.71
11f	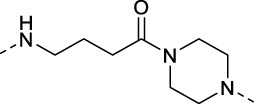	102.8	>100	20.19	>100
11g	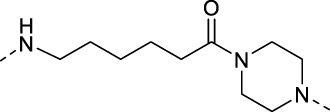	102.4	>100	19.18	82.90
11h	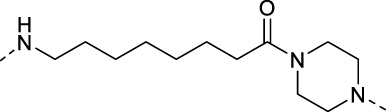	36.1	19.95	82.55	>100
S3I-201	—	46.94	111.4	19.02	109.0

### 3.5 SDL-1 inhibits cell cycle and induces cell apoptosis of gastric cancer cell line MKN1

**FIGURE 4:**
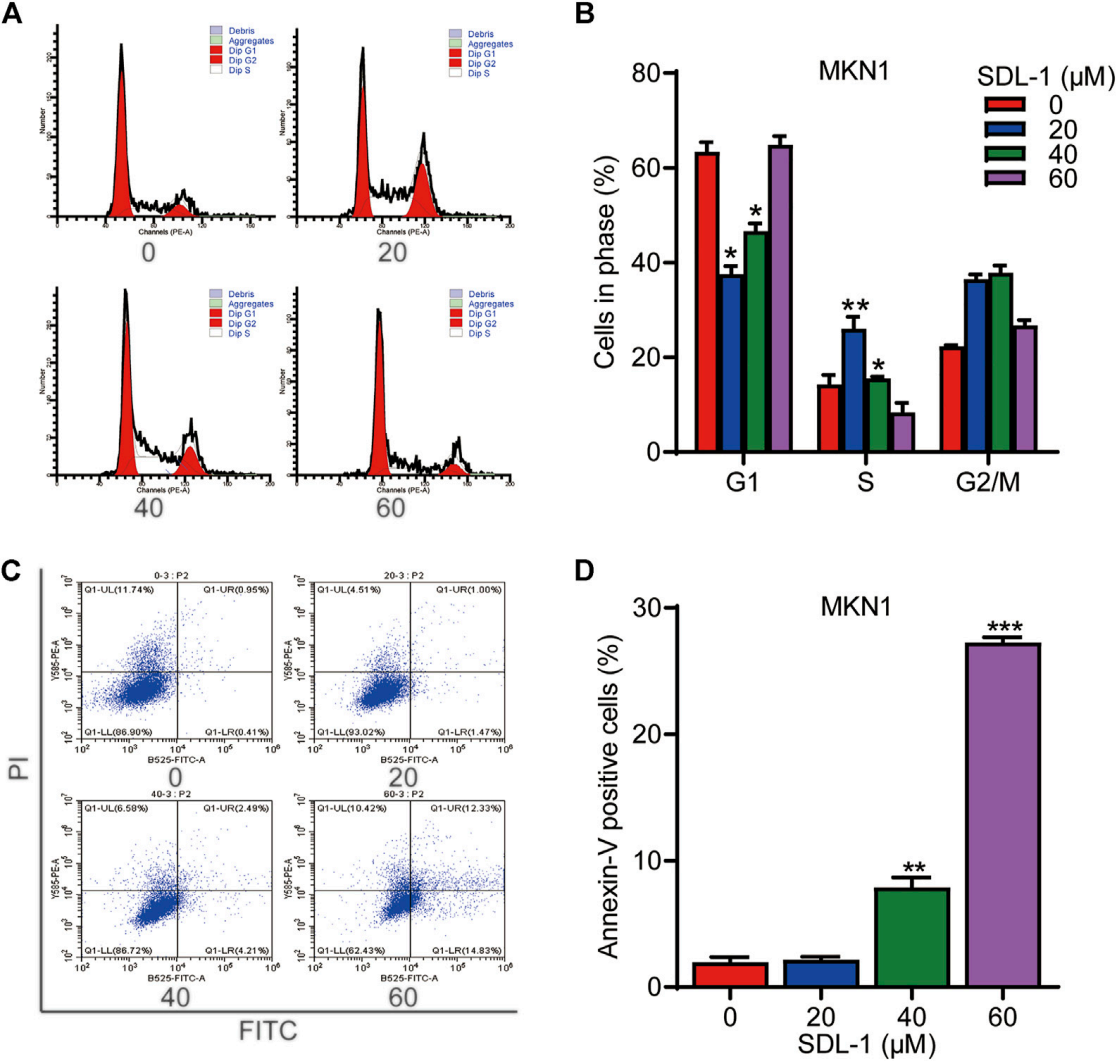
SDL-1 can block cell cycle of gastric cancer cell and induces apoptosis *in vitro*. **(A,B)** SDL-1 was treated for 24 h and fixed, followed by the propidium iodide/RNase analysis. **(C,D)** MKN1 cells were treated with SDL-1 at the indicated concentrations for 48 h, followed by the detection of apoptosis by FITC-Annexin V assay. Results are presented as mean ± SD. (*p < 0.05; **p < 0.01, ***p < 0.001).

To monitor the effect of SDL-1 on the cell cycle progression, we conducted the flow cytometric assay. The result shown that the proportions of G1, S phase and G2 cells changed remarkably among these four groups. At 20 μΜ, SDL-1 significantly increased the cell percentage in S phase from 14% to 26% in MKN1 cells (Figures 4A,B). Accordingly, we tested whether SDL-1 affect apoptosis of MKN1 cells. As shown in Figures 4C,D, we observed that cells did not undergo significant apoptosis under 20 μM SDL-1 treatment, and when the concentration reached 40 and 60 μM, the proportion of apoptotic cells were significantly increased in a concentration-dependent manner. The percentage of apoptotic cells in the highest concentration group was about 30%.

### 3.6 SDL-1 inhibits migration and invasion of gastric cancer cell line MKN1

**FIGURE 5:**
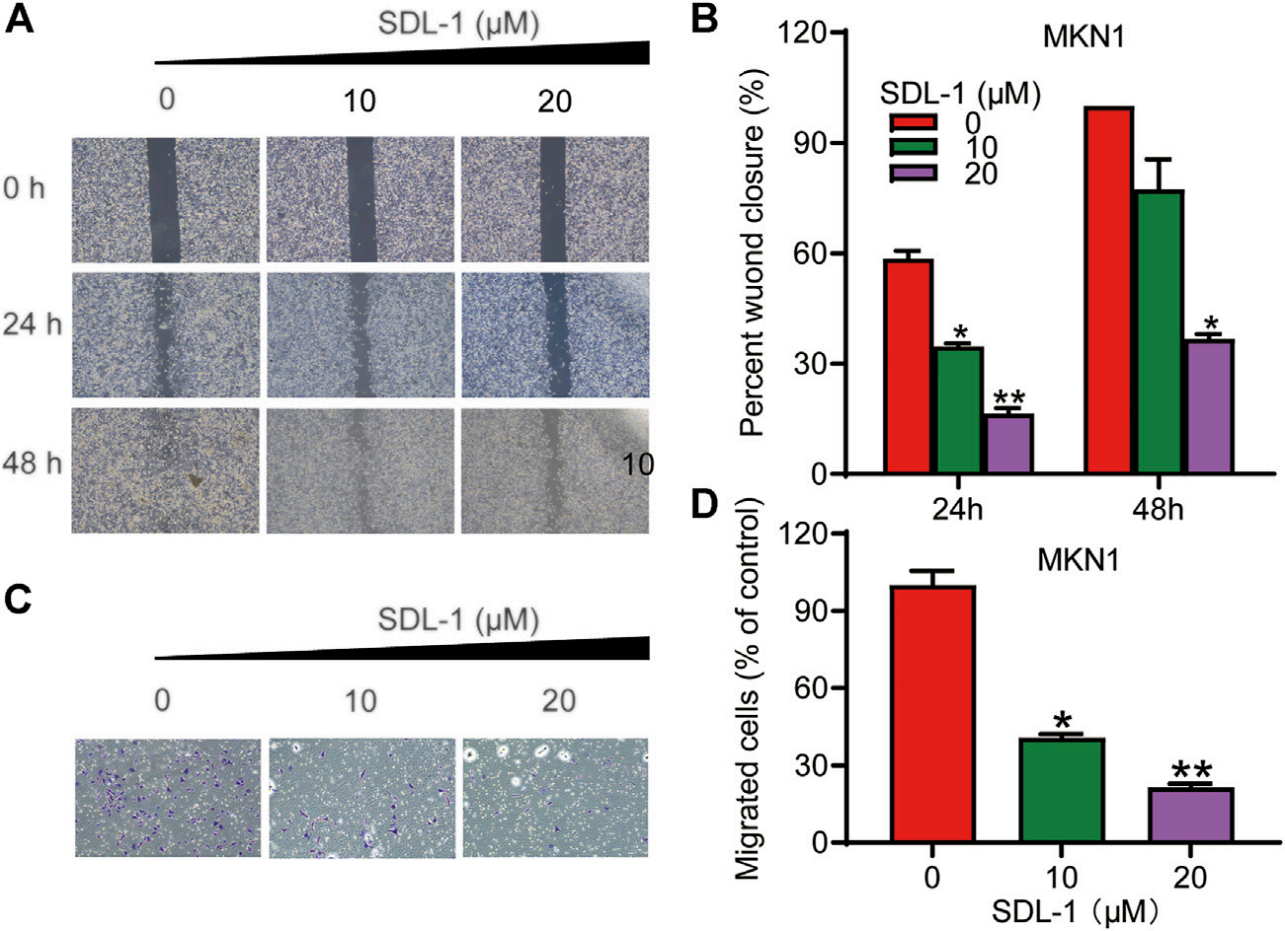
SDL-1 interfered with the invasion and metastasis of gastric cancer cell *in vitro*. **(A,B)** In the wound-healing assay, MKN1 cells were treated with SDL-1 at the specified concentration for 48 h, and the degree of cell healing was observed and recorded every 24 h **(C,D)** Transwell invasion assay was carried out in MKN1 cells treated with or without SDL-1. Results are presented as mean ± SD. (*p < 0.05; **p < 0.01, ***p < 0.001).

To examine the effects of SDL-1 on the migration and invasion of MKN1 cell line, we performed the wound-healing assay and transwell invasion assay. As shown in Figures 5A,B, MKN1 cells in the control group migrated into nearly 100 percent wounded area by 48 h, whereas migration in groups treated with specific concentrations (10 and 20 μM) of SDL-1 was effectively blocked in a concentration-dependent manner compared to the control group, especially after 20 μl of SDL-1 for 48 h, the mobility of MKN1 was 36.7% of the scratch area at 0 h. The results of the invasion assay in Figures 5C,D showed that the invasive ability of MKN1 cells in the group treated with a specific concentration of SDL-1 (10 and 20 μM) was significantly decreased in a concentration-dependent manner compared with the control group. The above experimental results indicated that SDL-1 significantly inhibited the migration and invasion ability of gastric cancer cell MKN1 in a dose-dependent manner, and the effect of inhibiting migration and migration may not be due to apoptosis after SDL-1 treatment, because no obvious apoptosis was observed under 20 μM SDL-1 treatment.

### 3.7 SDL-1 induces STAT3 protein degradation and decreases the phosphorylated STAT3 levels

**FIGURE 6:**
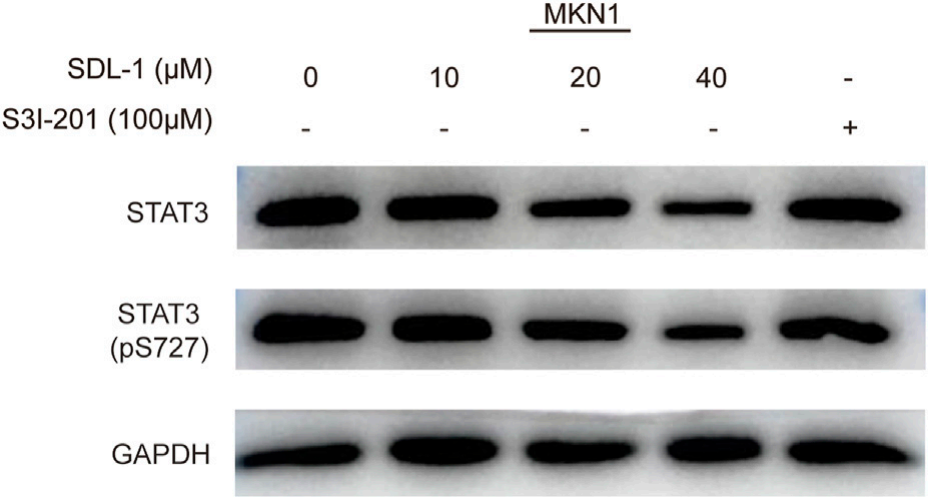
SDL-1 induced STAT3 protein degradation and decreased STAT3 phosphorylation levels *in vitro.* MKN1 cells were treated with the specified concentration of SDL-1 for 24 h, and the contents of various proteins in MKN1 cells were detected by Western blot.

We performed the Western blot assay to investigate the effect of SDL-1 on the STAT3 signaling pathway in gastric cancer cells. When cells were treated with SDL-1 at 10, 20, and 40 μM for 24 h, we found that SDL-1 concentration-dependently suppressed the protein level of total STAT3 and p-STAT3 (S727) in MKN1 cell line (Figure 6). S3I-201, an STAT3 inhibitor, was used as a positive control, which can inhibit the phosphorylation level of STAT3 without degrading the STAT3 (Figure 6). These data suggested that the anticancer effect caused by SDL-1 in gastric cancer cells was partly mediated by the STAT3 signaling pathway.

## 4 Discussion

**FIGURE 7:**
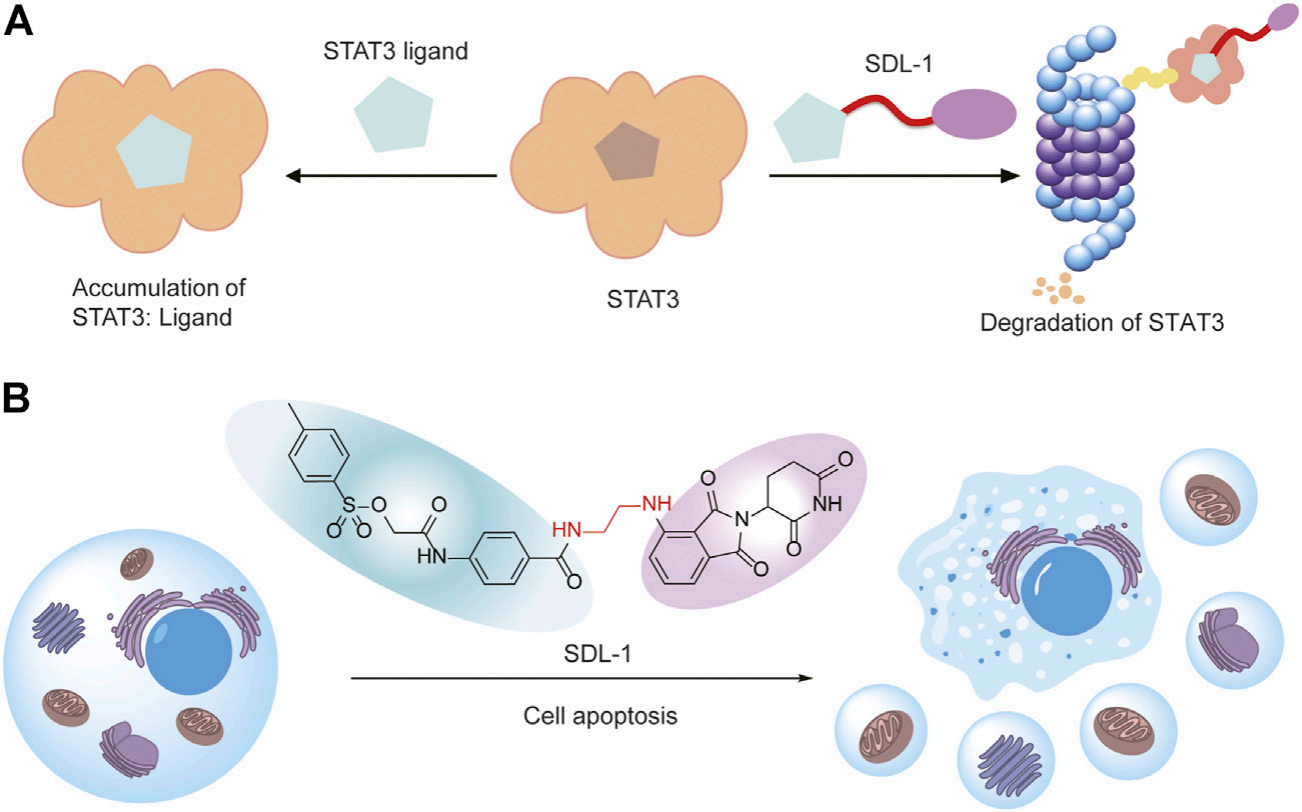
SDL-1 induces the formation of ternary complexes and leads to cell apoptosis. **(A)** The STAT3 ligand in SDL-1 binds to STAT3, and the E3 ubiquitin ligase ligand hijacks CRBN, transfers ubiquitin to STAT3 protein, and finally achieves ubiquitination and degradation of the target protein in the proteasome. **(B)** SDL-1 promotes the cell apoptosis of gastric cancer cells.

In this study, we found STAT3 is overexpressed and correlated with poor prognosis of gastric cancer. We designed and synthesized a series of S3I-201-based PROTACs for degrading STAT3 (Figure 7A). Then we evaluated their antitumor activity against gastric cancer cells, and we found SDL-1 presents the most promising anti-gastric cancer effects in HGC27, MGC803, AZ521, and MKN1 cells. We also demonstrated that SDL-1 significantly induces the cell cycle arrest, promotes the cell apoptosis, and inhibits migration and invasion of MKN1 cells. More importantly, we have confirmed that SDL-1 is a potent degrader of STAT3 and can affect the STAT3 signaling pathway by degrading STAT3 protein and inhibiting the STAT3 phosphorylation. Our data suggested that SDL-1 as a potent STAT3 degrader may be a potential anti-gastric cancer drug candidate, and PROTAC targeting STAT3 may be a new and effective gastric cancer therapeutic strategy (Figure 7B).

Recently, Wang’s group reported a potent and effective STAT3 degrader SD-36, which selectively induced rapid STAT3 degradation at low nanomolar concentrations in leukemia and lymphoma cells, and achieved nanomolar cytostatic activity. In addition, SD-36 caused complete degradation of STAT3 protein in xenograft tumor tissues and normal mouse tissues (Bai et al., 2019; Li, Guo, Wang, Chen, & Mei, 2020; Zhou et al., 2019). Another study developed a Napabucasin-based STAT3 PROTAC XD2-149, which inhibited STAT3 signaling in pancreatic cancer cell lines, but did not induce proteasome-dependent STAT3 degradation, suggesting that targeting STAT3 degradation remains challenging (Hanafi, Chen, & Neamati, 2021). S3I-201 has been reported to selectively inhibit STAT3 DNA-binding activity *in vitro* with an IC_50_ value of 86 μM (Siddiquee et al., 2007). In this study, we report the design and synthesis of a series of STAT3 PROTACs based on S31-201 against gastric cancer for the first time, providing a possible direction for the clinical treatment of gastric cancer.

There are still some limitations of our study. The efficacy of SDL-1 as a STAT3 degrader for the treatment of other human cancers or conditions in which STAT3 plays an important role warrants further investigation. In addition, the pharmacological effects, pharmacokinetic/pharmacodynamic (PK/PD) properties, and host toxicity of SDL-1 should be evaluated in multiple xenograft mouse models *in vivo.* Furthermore, although SDL-1 was designed based on S3I-201, the binding affinity and exact binding site remain to be determined.

In conclusion, this study demonstrates that SDL-1 is a potent STAT3 degrader, induces STAT3 protein degradation *in vitro*, and inhibits gastric cancer growth and metastasis. These results suggest that targeting STAT3 with PROTAC may provide a new avenue for gastric cancer therapy.

## Data Availability

The original contributions presented in the study are included in the article/supplementary material, further inquiries can be directed to the corresponding authors.
